# Breaking the Cycle: Impact of Physical Activity on Sleep Disorders in Autism—A Five-Year Longitudinal Analysis

**DOI:** 10.3390/children13010048

**Published:** 2025-12-30

**Authors:** Eman A. Toraih, Jason Zeleny, Carol Sames, Andrew Craig, Catherine Hagearty-Mattern, Sierra Coyle, Amanda Lois, Rami M. Elshazli, Hani Aiash

**Affiliations:** 1 Department of Cardiovascular Perfusion, College of Health Professions, Upstate Medical University, Syracuse, NY 13210, USA; aiashh@upstate.edu; 2 Medical Genetics Unit, Department of Histology and Cell Biology, Suez Canal University, Ismailia 41522, Egypt; 3 Department of Pediatrics, Norton College of Medicine, SUNY Upstate Medical University, Syracuse, NY 13210, USA; zelenyj@upstate.edu (J.Z.); craiga@upstate.edu (A.C.); 4 Department of Behavior Analysis Studies, College of Health Professions, SUNY Upstate Medical University, Syracuse, NY 13210, USA; 5 Department of Physical Therapy Education, College of Health Professions, Upstate Medical University, Syracuse, NY 13210, USA; samesc@upstate.edu (C.S.); loisa@upstate.edu (A.L.); 6 Laboratory Sciences, Department of Clinical, College of Health Professions, Upstate Medical University, Syracuse, NY 13210, USA; hageartc@upstate.edu; 7 Department of Respiratory Therapy Education, College of Health Profession, Upstate Medical University, Syracuse, NY 13210, USA; coyles@upstate.edu; 8 Department of Surgery, School of Medicine, Tulane University, New Orleans, LA 70112, USA; 9 Biochemistry and Molecular Genetics Unit, Department of Basic Sciences, Faculty of Physical Therapy, Horus University-Egypt, New Damietta 34517, Egypt; 10 Department of Biological Sciences, Faculty of Science, New Mansoura University, New Mansoura City 35742, Egypt

**Keywords:** autism spectrum disorder, sleep disorders, physical activity, children, adolescents, electronic health records, retrospective cohort study, prospective validation, implementation science

## Abstract

Background/Objectives: Sleep disorders represent a significant health burden among children and adolescents with autism spectrum disorder (ASD), affecting their core symptoms, behavior, and quality of life. While physical activity has shown promise in managing sleep disorders in the general pediatric population, its effectiveness for children and adolescents with ASD remains understudied. Methods: This retrospective cohort study analyzed electronic health records from 132 healthcare organizations, examining 155,860,529 individuals to determine sleep disorder prevalence in ASD populations and evaluate the impact of physical activity interventions. We identified 248,940 children and adolescents with ASD aged 5–18 years, of whom 38,976 had documented sleep disorders. Propensity score matching was performed to compare patients with ASD and sleep disorders who received physical activity interventions with matched controls. Primary outcomes included sleep disorder resolution and medication utilization changes at 1- and 5-year follow-up. Bonferroni correction was applied to secondary analyses to account for multiple comparisons. Results: The prevalence of sleep disorders was markedly higher in children and adolescents with ASD (19.25%) compared to non-ASD peers (3.37%), with risk ratios escalating from childhood (RR = 5.34, 95% CI: 5.28–5.40) to adolescence (RR = 6.12, 95% CI: 6.05–6.19). After matching, 3709 patients were included in each group. Physical activity interventions were associated with significantly higher sleep disorder resolution at 1 year (−59.9% vs. −5.05%, *p* = 0.001) and sustained benefit at 5 years (−49.83% vs. +7.26%, *p* = 0.001). After Bonferroni correction, improvement in sleep apnea at 1 year remained statistically significant (−62.26% vs. +9.39%, Bonferroni-adjusted *p* = 0.040). Improvements in parasomnia and insomnia did not survive correction and were considered exploratory. Age emerged as a key effect modifier: adolescents (12–18 years) demonstrated sustained improvements in overall sleep outcomes at both 1- and 5-year follow-up that met Bonferroni-corrected thresholds, whereas younger children (5–11 years) showed limited and inconsistent responses. Among comorbidity groups, anxiety-comorbid patients exhibited the strongest overall improvement (−58.7% vs. −12.4%, *p* < 0.01), while reductions in amphetamine use and changes in melatonin prescribing patterns should be interpreted as exploratory findings requiring prospective confirmation. Conclusions: This large-scale observational study suggests structured physical activity interventions are associated with sustained improvements in overall sleep disorders among children and adolescents with ASD. While subtype- and subgroup-specific associations were observed, many attenuate after multiple comparison adjustments, highlighting the need for cautious interpretation. Findings support exploring physical activity in comprehensive care plans, with prospective randomized trials needed to confirm causality, optimize protocols, and address multiplicity.

## 1. Introduction

Sleep disorders represent a significant health burden among children and adolescents with autism spectrum disorder (ASD). Research indicates that between 40–80% of children and adolescents with autism struggle with sleep issues, a substantially higher rate than the 20–30% observed in typically developing children and adolescents [1,2,3,4,5]. These sleep disturbances manifest in various forms, including difficulties with sleep onset, maintenance, and circadian rhythm disruptions, often persisting despite standard interventions [6]. Sleep problems may also extend far beyond nighttime hours, potentially worsening core symptoms of ASD, increasing behavioral challenges, affecting cognitive abilities, and diminishing quality of life for both children or adolescents and their caregivers [3,4,7].

Traditionally, clinicians have relied on pharmacological treatments to treat sleep disorders in ASD children. However, concerns related to side effects and long-term medication have raised interest in alternative approaches [8,9,10]. Regular physical activity has emerged as a promising intervention strategy, with preliminary evidence suggesting its potential to help regulate sleep–wake cycles, reduce time to fall asleep, and enhance overall sleep quality in typically developing individuals [10,11,12,13]. Yet, few studies have thoroughly examined whether participation in structured physical activity programs is associated with better sleep outcomes in children with ASD.

The interplay between physical activity and sleep in ASD children and adolescents presents complexities [14]. Sleep difficulties often change as children and adolescents age, while common co-occurring conditions such as anxiety and ADHD can affect both sleep patterns and willingness to participate in physical activities [1,4,15,16]. The inherent characteristics of ASD, especially sensory sensitivities and motor coordination difficulties, may create barriers to engaging physical activities [1,17]. Given the tremendous variation in how ASD manifests across individuals, physical activity interventions likely produce different outcomes depending on specific patient characteristics [1,18]. Understanding these intricate relationships is crucial to developing effective, personalized interventions.

To our knowledge, most existing research in this area is limited by small sample sizes, short follow-up periods, and insufficient consideration of important confounding factors such as comorbid conditions and medication use. To address these limitations, our study examines the association between structured physical activity interventions and sleep outcomes using electronic health records from a large cohort of children and adolescents with ASD. We aim to determine the prevalence of diagnosed sleep disorders in this population, evaluate whether participation in structured physical activity programs is associated with improved resolution of sleep disorders, and identify how these effects might vary based on age, specific sleep disorder type, and presence of comorbidities. This large-scale approach enables us to examine these relationships comprehensively while controlling for crucial clinical and demographic factors that might influence results.

## 2. Materials and Methods

### 2.1. Data Source

This retrospective cohort study utilized data from the TriNetX global health research network, a federated network of electronic health records (EHR) from healthcare organizations (HCOs). The study period spanned two decades with data collected from 132 participating HCOs (accessed 29 November 2024).

### 2.2. Study Population and Design

The study was conducted in two phases addressing distinct research questions. For the prevalence analysis, we identified children and adolescents aged 5–18 years with and without ASD diagnoses using ICD-10 codes (F84.0). Sleep disorders were identified using ICD-10 code G47. The prevalence of sleep disorders was calculated for the overall population and stratified by age groups (5–11 and 12–18 years).

For the intervention analysis, we focused on patients with concurrent ASD and sleep disorder diagnoses (Supplementary Table S1). Patients were excluded if they had severe comorbidities that could significantly impact physical activity or sleep patterns, including cerebral palsy, severe intellectual disability, chromosomal abnormalities, congenital heart disease, or active malignancy (Supplementary Table S2). Physical activity interventions after the diagnosis of ASD and sleep disorders were identified using multiple standardized coding systems, including CPT codes (97110, 97112, 97116, 97530, 97150), ICD-10-PCS procedure codes (F07M6YZ, F07M6ZZ, F07M6FZ), HCPCS codes (S9451), and SNOMED CT codes (66500008, 91251008, 229065009, 819961005) for physical medicine procedures and exercise therapy as detailed in Supplementary Table S1. These codes predominantly represent structured, clinician-initiated physical activity delivered within healthcare or rehabilitation settings rather than informal lifestyle counseling or unsupervised activity recommendations.

### 2.3. Outcomes Assessment

Primary outcomes included sleep disorder resolution (defined as the absence of diagnosis codes in follow-up), time to resolution (measured in days from intervention initiation), and changes in sleep medication utilization. Sleep disorders were classified using specific ICD-10 codes, including insomnia (G47.0), hypersomnia (G47.1), circadian rhythm disorders (G47.2), sleep-related breathing disorders (G47.3), narcolepsy and cataplexy (G47.4), parasomnia (G47.5), and other sleep disorders (G47.8, G47.9) (Supplementary Table S3).

### 2.4. Statistical Analysis

For the prevalence analysis, we calculated proportions with 95% confidence intervals and computed risk ratios to compare sleep disorder prevalence between ASD and non-ASD populations, both overall and stratified by age groups. In the intervention analysis, we employed propensity score matching (1:1 with nearest neighbor method) to create comparable groups of children and adolescents with ASD and sleep disorders who did and did not receive physical activity interventions. Matching variables included demographics (age, sex, race/ethnicity), comorbidities (asthma, overweight/obesity, mood disorders, anxiety disorders, intellectual disabilities, ADHD, epilepsy, and musculoskeletal disorders), and sleep-related medication use (melatonin, sedatives/hypnotics, methylphenidate, amphetamines) within 6 months of index event.

Resolution rates were calculated using the formula: “Resolution Rate = [(Cases at follow-up − Initial cases)/Initial cases] × 100%”. Negative values indicate improvement (reduction in cases); positive values indicate worsening (increase in cases). Time-to-event analysis was conducted using Cox proportional hazards models to estimate the association between physical activity interventions and sleep disorder resolution. Analyses were adjusted for relevant covariates with assessment of proportional hazards assumptions. Secondary analyses included stratification by age groups, sleep disorder subtypes, and presence of key comorbidities. Given the multiple statistical comparisons across sleep disorder types, age groups, and comorbidity subgroups, we applied Benjamini–Hochberg false discovery rate (FDR) correction to control for Type I errors. Statistical significance was set at *p* < 0.05, and all analyses were performed using TriNetX built-in analytic tools.

## 3. Results

### 3.1. Prevalence of Sleep Disorders

Our analysis encompassed 155,860,529 individuals across 132 healthcare organizations. Within this population, 588,517 patients (0.37%) had documented ASD diagnoses. For the prevalence analysis, we identified 344,244 individuals with ASD diagnoses aged 5–18 years. Notably, 66,372 of these children and adolescents (19.25%) had documented sleep disorders—a rate substantially higher than the 3.37% (666,416 of 19,757,552) prevalence observed in age-matched children and adolescents without ASD.

Age-stratified analyses demonstrated increasing sleep disorder prevalence with age in the ASD population. Among children aged 5–11 years, 18.46% (33,381 of 180,784) with ASD had diagnosed sleep disorders, compared to 3.45% (321,379 of 9,301,042) in their non-ASD peers. The prevalence increased in adolescents aged 12–18 years, reaching 20.18% (32,991 of 163,460) for those with ASD, versus 3.29% (345,037 of 10,456,510) in adolescents without ASD, as shown in Table 1.

Risk analyses indicated that patients with ASD had a 246% increased risk of sleep disorders compared to those without ASD (RR = 3.46, 95% CI: 3.44–3.48). This disparity was more pronounced in age-stratified analyses, with children aged 5–11 years showing approximately five times the risk (RR = 5.34, 95% CI: 5.28–5.40) and adolescents aged 12–18 years demonstrating a six-fold increased risk (RR = 6.12, 95% CI: 6.05–6.19) compared to their non-ASD peers.

### 3.2. Impact of Physical Activity Intervention

**Figure 1:**
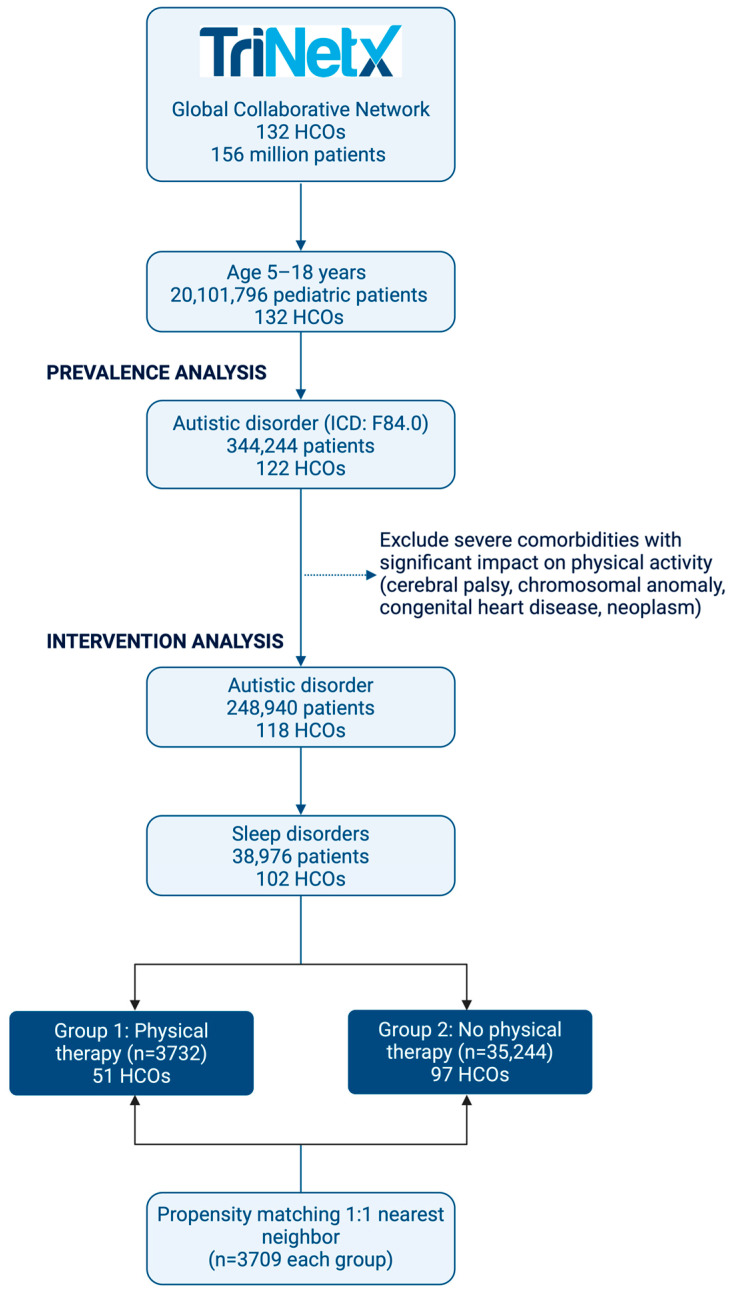
Flow diagram of cohort selection for evaluating physical activity interventions in children and adolescents with autism spectrum disorder and sleep disorders. HCOs, healthcare organizations.

For the intervention analysis phase, we applied rigorous exclusion criteria resulting in 248,940 pediatric ASD patients from 118 healthcare organizations. Among these, 38,976 patients had documented sleep disorders, with 3732 patients receiving physical activity interventions and 35,244 who did not, as shown in Figure 1.

After propensity score matching, 3709 patients were included in each group, with balanced demographic characteristics and comorbidity profiles. The matched cohorts showed no significant differences in key variables, with both groups comprising predominantly male patients (71.9% in the intervention group, 72.8% in the control group) and children aged 5–11 years (62.2% and 63.1%, respectively), as shown in Table 2.

**Figure 2:**
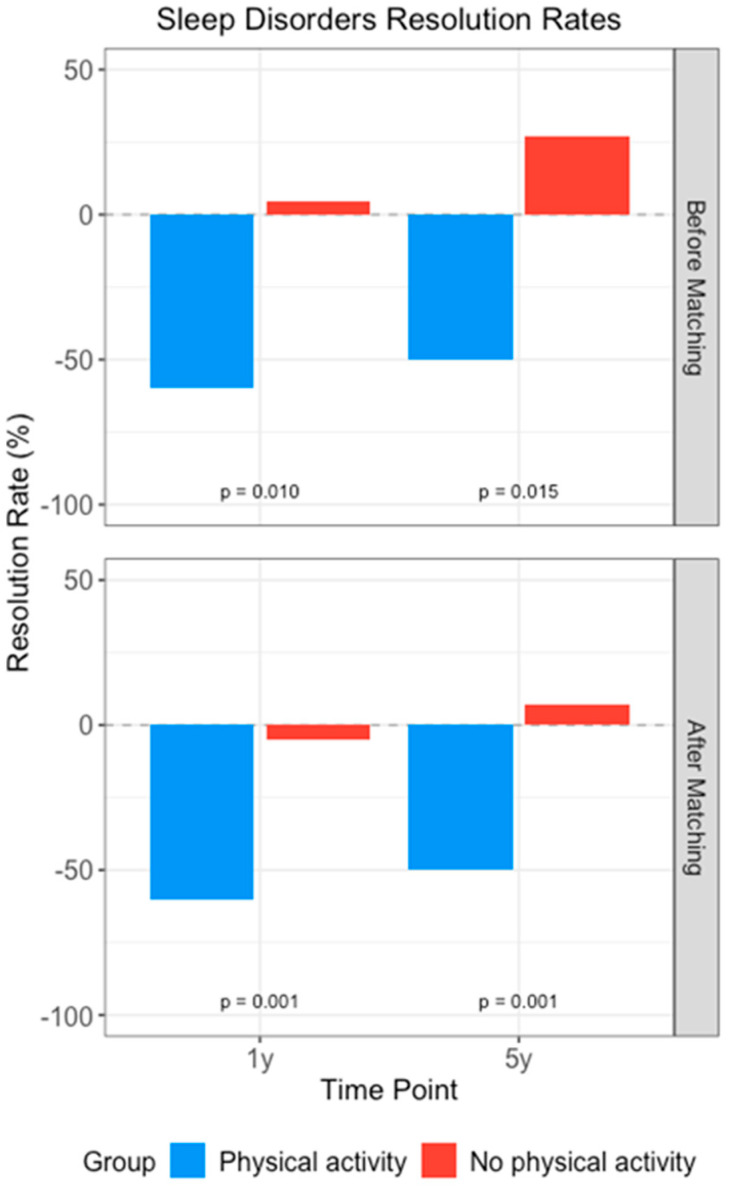
Resolution rates of sleep disorders in children and adolescents with autism spectrum disorder by physical activity intervention. Bars represent resolution rates (%) at 1-year and 5-year follow-up for physical activity (blue) and no physical activity (red) groups, before and after propensity score matching. *p*-values of each time point comparison are shown. Negative values indicate improvement from baseline; Positive values indicate worsening from baseline.

Higher sleep disorder resolution rates were observed in the physical activity group. At 1-year follow-up, the number of affected individuals decreased to 1484 in the intervention group while increasing to 3522 in the control group (*p* = 0.001). The intervention group, after matching, achieved a resolution rate of −59.9% (indicating 59.9% improvement from baseline; cases decreased from 3709 to 1484), compared to −5.05% in the control group. This improvement persisted at 5 years, with a −49.83% resolution rate in the intervention group contrasting with a 7.26% increase in sleep disorders in the control group (*p* = 0.001), Figure 2. Time-to-event analysis indicated an association with reduced risk of persistent sleep disorders (HR = 0.86, 95% CI: 0.80–0.92 at 1 year; HR = 0.88, 95% CI: 0.83–0.94 at 5 years). In other words, the intervention group’s engagement in structured physical activity appears correlated with a reduction in the risk of negative sleep outcomes over time.

Analysis of specific sleep disorder subtypes revealed differential responses to physical activity interventions, Table 3 and Supplementary Table S4. The most pronounced improvements at 1-year follow-up were observed in parasomnia (−83.33% vs. −1.79%, *p* = 0.016), circadian rhythm sleep disorders (−71.65% vs. −19.12%, *p* = 0.035), and sleep apnea (−62.26% vs. +9.39%, *p* = 0.004). Parasomnia demonstrated the most durable response, maintaining significant improvements at both 1 year and 5 years (−63.89% vs. −14.29%, *p* = 0.048), while sleep apnea-maintained benefits through the 5-year follow-up (−47.69% vs. +8.82%, *p* = 0.027). After applying Bonferroni correction (α = 0.005), sleep apnea at 1-year follow-up emerged as the most robust finding, with the intervention group demonstrating substantial improvement (Bonferroni-adjusted *p* = 0.040).

### 3.3. Age-Stratified Analysis: Developmental Differences in Treatment Response

Age-stratified analysis highlighted distinct characteristics between child and adolescent cohorts. The younger cohort (5–11 years, n = 2682) was predominantly male (76.9%) with notable rates of ADHD (53.6%) and anxiety disorders (39.7%), Supplementary Table S5. The adolescent cohort (12–18 years, n = 1634) showed a more balanced gender distribution (38.3% female) and higher rates of psychiatric comorbidities, including anxiety disorders (73.9%), ADHD (60.7%), and mood disorders (49.4%), Supplementary Table S6.

Analysis of sleep disorder resolution rates uncovered age-specific responses to physical activity interventions. In the younger cohort (5–11 years), overall sleep disorders showed significant improvement at one year, with a resolution rate of −56.30% in the physical activity group compared to −7.05% in the control group (*p* = 0.036, Bonf *p* = 0.43), which did not meet Bonferroni-corrected significance. Specific sleep disorder subtypes similarly showed exploratory improvements: insomnia (−52.00% vs. −27.43%, *p* = 0.025, Bonf *p* = 0.30), circadian rhythm sleep disorders (−50.00% vs. −13.64%, *p* = 0.041, Bonf *p* = 0.49), and parasomnia (−73.33% vs. −16.15%, *p* = 0.036, Bonf *p* = 0.43). At 5-year follow-up, improvements in the younger cohort were largely attenuated. Overall sleep disorder resolution showed convergence between groups (−60.61% intervention vs. −49.92% control, *p* = 0.49). Circadian rhythm sleep disorders showed the most sustained improvement at 5-years (−59.62% vs. −16.36%, *p* = 0.007, Bonf *p* = 0.084), approaching but not meeting Bonferroni significance. No other findings in the younger cohort met corrected significance thresholds, as shown in Table 4.

The adolescent group (12–18 years) yielded more pronounced responses to physical activity interventions. Overall sleep disorder resolution rates at one year follow-up were markedly higher (−82.91% vs. −21.00%, *p* = 0.003, Bonf *p* = 0.036), with this improvement maintained at five years (−80.06% vs. −7.14%, *p* < 0.001, Bonf *p* = 0.006). Sleep apnea exhibited the most pronounced responses in adolescents, with considerable improvements in both one year (−80.42% vs. −14.46%, *p* = 0.036, Bonf *p* = 0.43) and five years (−81.47% vs. +20.46%, *p* < 0.001, Bonf *p* = 0.006). Insomnia also maintained improvement, with significant resolution rates at both one year (−68.77% vs. −22.43%, *p* = 0.032, Bonf *p* = 0.38) and five years (−60.89% vs. −36.45%, *p* = 0.006, Bonf *p* = 0.07). Hypersomnia, circadian rhythm sleep disorders, and parasomnia did not show significant differential responses in the adolescent group, as shown in Table 4.

### 3.4. Nested Analysis by Comorbidity

Analysis of the ADHD subgroup (n = 4896) revealed substantial comorbidity burden and distinct medication patterns. After matching, these patients exhibited high rates of anxiety disorders (59.1%) and musculoskeletal disorders (44.4%). Sleep medication use was notable, with 39.3% prescribed sedatives/hypnotics and 32.6% using melatonin. Most patients (60.2%) were in the younger age group (5–11 years), Supplementary Table S7.

The anxiety disorder cohort (n = 4010) highlighted significant overlap with other conditions, particularly ADHD (68.7%) and musculoskeletal disorders (49.7%). Age distribution was relatively balanced between children and adolescents (49.9% vs. 41.3%). Sleep medication utilization patterns included substantial use of sedatives/hypnotics (41.8%) and melatonin (31%), Supplementary Table S8.

The epilepsy cohort (n = 880), though smaller, displayed unique characteristics, notably showing the highest rate of sedative/hypnotic use (80%) among all subgroups. These patients exhibited substantial psychiatric comorbidity, with ADHD affecting 55.2% and anxiety disorders present in 46.6% of cases. The population was predominantly younger (58%) and showed balanced gender representation (28.9% female), Supplementary Table S9.

Comorbidity-specific analyses indicated varying responses to physical activity interventions across different sleep disorder subtypes and patient groups. The anxiety group consistently showed the strongest overall response to intervention, with general sleep disorder resolution rates of −58.7% vs. −12.4% at one year and −51.2% vs. +3.9% at five years (*p* < 0.01). The ADHD group exhibited intermediate response levels (−54.2% vs. −8.3% in one year, *p* < 0.01), while the epilepsy group showed more modest yet still significant benefits (−45.6% vs. −2.8% at one year, *p* < 0.05), Table 5.

Analysis of specific sleep disorders within these comorbidity groups uncovered distinctive patterns. Parasomnia showed the most dramatic improvements with physical activity across all groups, with resolution rates ranging from −75% to −85% at one year. Sleep apnea demonstrated a unique response pattern, with the control group showing immediate symptoms of worsening after one year, while the intervention group achieved significant improvement. In contrast, hypersomnia showed the smallest between-group differences and failed to reach statistical significance across all comorbidity groups, Table 5.

### 3.5. Medication Utilization Changes

Analysis of medication utilization patterns highlighted distinct impacts across different medications and comorbidity groups. In the overall cohort (N = 7418), melatonin persistence differed significantly between groups. While both the physical activity and control groups showed absolute decreases in melatonin use (intervention: 28.9%→17.1%, −11.80%; control: 27.8%→14.5%, −13.30%), the intervention group demonstrated slower discontinuation (HR for discontinuation: 1.21, 95% CI: 1.078–1.357, *p* = 0.001, Bonf *p* = 0.003), indicating greater retention of melatonin use in the intervention group over follow-up.

Amphetamine use decreased in both groups, with the intervention group showing a more pronounced reduction (intervention: 19.3%→15.0%, −4.3%; control: 19.4%→ 16.9%, −2.5%; HR: 0.865, 95% CI: 0.771–0.969, *p* = 0.024); however, this finding does not meet Bonferroni-corrected significance threshold (Bonf *p* = 0.072) and should be interpreted cautiously as an exploratory observation. Methylphenidate utilization showed no significant difference between groups (intervention: 24.9%→19.2%, −5.7% vs. control: 25.0%→20.5%, −4.5%; HR: 0.928, 95% CI: 0.838–1.028, *p* = 0.17), Table 6.

Comorbidity-specific medication analyses revealed that ADHD patients (n = 4896) and anxiety patients (n = 4010) showed the strongest positive associations with melatonin use (HR: 1.303 and 1.334, respectively, *p* = 0.001, Bonf *p* = 0.009). The epilepsy group (n = 880) showed borderline reduction in amphetamine use (*p* = 0.054), while other medications showed no significant changes in this subgroup, Table 7.

## 4. Discussion

This large-scale retrospective study provides observational evidence suggesting an association between structured physical activity interventions and improved sleep outcomes among children and adolescents with autism spectrum disorder. Analyzing data from over 155 million individuals across 132 healthcare organizations, we identified three principal findings: (1) sleep disorders are markedly more prevalent in pediatric ASD populations compared to neurotypical peers, with risk ratios escalating from childhood (RR = 5.34) to adolescence (RR = 6.12); (2) physical activity interventions were associated with substantial sleep disorder resolution rates (−59.9% at one year, −49.83% at five years) that varied significantly by age group, sleep disorder subtype, and psychiatric comorbidity profile; and (3) these associations persisted after rigorous propensity score matching and were accompanied by favorable medication utilization patterns. These findings have important implications for clinical practice and highlight critical directions for future research.

### 4.1. Sleep Disorder Burden in Pediatric ASD Populations

The markedly higher prevalence of sleep disorders in children and adolescents with ASD (19.25%) compared to non-ASD peers (3.37%) aligns with previous estimates suggesting that 40–80% of autistic youth experience sleep difficulties [7,19,20]. However, our operational definition based on diagnostic codes likely substantially underestimates true prevalence, as many sleep disturbances in ASD may be managed through behavioral strategies without formal diagnosis or may remain undiagnosed due to diagnostic challenges. Polysomnography, the gold standard for confirming sleep disorders, requires continuous monitoring in unfamiliar laboratory environments—a context that significantly challenges autistic individuals due to sensory sensitivities and anxiety [21]. Clinicians must therefore frequently develop care plans based on clinical presentation rather than objective sleep architecture data, potentially leading to underdiagnosis and undertreatment.

Our observation of progressive risk escalation from childhood (RR = 5.34) to adolescence (RR = 6.12) represents a novel finding with important clinical implications. This age-dependent pattern may reflect the interaction between ASD-related sleep vulnerabilities and the neurobiological changes characteristic of adolescence, including circadian phase delay, increased sleep homeostatic pressure, and heightened sensitivity to environmental stressors [22,23,24]. Additionally, autistic adolescents face escalating academic demands, reduced structured support compared to childhood, and increasing awareness of social differences—factors that may compound sleep difficulties. The heterogeneity in sleep disorder subtypes observed across our cohort (insomnia: 42.8%, sleep apnea: 23.4%, circadian rhythm disorders: 15.2%, parasomnia: 12.6%) underscores the need for comprehensive sleep assessment and subtype-specific intervention strategies rather than one-size-fits-all approaches [25,26].

### 4.2. Physical Activity Interventions and Sleep Outcomes

Physical activity interventions demonstrated substantial associations with sleep disorder resolution, with rates of −59.9% at one year and −49.83% at five years in the intervention group compared to −5.05% and −7.26% in controls, respectively. The durability of these associations across a five-year observational period challenges conventional assumptions that sleep disorders in ASD represent invariably chronic conditions resistant to intervention. However, interpretation requires careful consideration of our study design limitations [27,28]. Our dataset captures billing codes for formal healthcare-delivered physical activity interventions but provides no information regarding critical dose parameters—including modality (aerobic, resistance, aquatic, recreational), intensity, frequency, duration, total session number, or adherence rates. This limitation prevents us from establishing dose–response relationships or identifying optimal intervention characteristics, essential for clinical translation.

The substantial heterogeneity within our intervention cohort—likely encompassing activities ranging from structured physical therapy to general exercise recommendations—may obscure stronger associations that might emerge with standardized protocols. This heterogeneity simultaneously limits interpretability and suggests real-world effectiveness across diverse intervention approaches. Future randomized controlled trials should systematically vary intervention parameters (frequency: 2–5 sessions/week; duration: 30–60 min/session; modality: aerobic vs. resistance vs. combined; setting: individual vs. group) to identify optimal prescriptions for specific patient profiles. Based on the physical activity codes captured in our dataset (Supplementary Table S1), we recommend that future trials prioritize: (1) moderate-intensity aerobic activities with rhythmic, repetitive movements (walking, cycling, swimming) that may enhance circadian entrainment; (2) activities accommodating sensory sensitivities and motor coordination challenges; and (3) interventions deliverable in both clinical and community settings to maximize accessibility and sustainability.

### 4.3. Age-Specific Response Patterns and Mechanisms

The differential response patterns across age groups provide particularly compelling evidence for developmental moderation of intervention effects. Adolescents (12–18 years) demonstrated markedly superior outcomes compared to younger children (5–11 years), with overall resolution rates of −82.91% versus −56.30% at one year and sustained five-year benefits (−80.06% vs. −60.61%). Sleep disorder-specific analyses revealed especially pronounced adolescent advantages for sleep apnea (−80.4% vs. −35.2% at one year; −81.4% vs. −45.6% at five years) and insomnia (−68.7% vs. −52.0% at one year). While our dataset does not permit direct examination of whether adolescents received different intervention types or doses, we propose several developmental mechanisms that may contribute to enhanced adolescent responsiveness independent of intervention characteristics [29,30].

First, improved motor coordination in adolescence may allow more effective engagement with physical activities, particularly those requiring sustained rhythmic movement or complex motor sequences. Second, greater cognitive capacity for understanding intervention rationale and maintaining exercise routines may enhance adherence and self-directed practice. Furthermore, adolescence represents a period of heightened circadian plasticity, with the biological clock demonstrating increased sensitivity to environmental timing cues [31,32]. Physical activity, particularly when performed at consistent times of day, may serve as a powerful zeitgeber (time-giver) that entertains circadian rhythms more effectively during this developmental window. Additionally, the neurobiological changes in adolescence—including ongoing prefrontal cortex maturation and reorganization of sleep–wake regulatory systems—may create opportunities for activity-induced neuroplasticity that are less accessible in younger children. These hypothesized mechanisms require empirical validation through studies incorporating objective measures of motor function, circadian phase (via dim light melatonin onset), and sleep architecture (via polysomnography or actigraphy) [33,34].

### 4.4. Sleep Disorder Subtype-Specific Responses

Analysis of specific sleep disorder subtypes revealed mechanistically informative response patterns. Physical activity demonstrated particularly dramatic associations with parasomnia resolution (−83.33% vs. −1.79% at one year; −63.89% vs. −14.29% at five years), sleep apnea (−62.26% vs. +9.39% at one year; −47.69% vs. +8.82% at five years), and circadian rhythm disorders (−71.65% vs. −19.12% at one year). These differential responses suggest that physical activity may beneficially modulate multiple sleep pathophysiological mechanisms, including arousal regulation (parasomnia), respiratory control and upper airway patency (sleep apnea), and circadian phase alignment (circadian rhythm disorders). In contrast, hypersomnia showed limited differential response, suggesting distinct underlying mechanisms potentially requiring complementary interventions such as scheduled napping, bright light therapy, or stimulant medications [35,36].

The substantial improvement in sleep apnea warrants particular attention given the elevated obesity prevalence in autistic populations [37]. Although obesity was balanced between groups through propensity score matching (18.4% intervention vs. 17.2% control, *p* = 0.16), we lack serial anthropometric measurements to determine whether sleep apnea improvements correlate with weight reduction. Physical activity represents a primary intervention for weight management, and the observed sleep apnea resolution may be partially mediated through reductions in adipose tissue burden on upper airway structures. However, physical activity may also improve sleep apnea through weight-independent mechanisms, including enhanced respiratory muscle tone, reduced systemic inflammation, and improved autonomic nervous system regulation. Future prospective studies should incorporate detailed anthropometric assessments (body mass index, waist circumference, bioelectrical impedance analysis) at baseline and follow-up intervals to parse weight-dependent from weight-independent pathways of benefit.

### 4.5. Proposed Biological Mechanisms

We propose that the observed associations between physical activity and sleep improvement may reflect multiple interacting biological mechanisms, though these hypotheses remain speculative pending empirical validation. First, regular physical activity may serve as a powerful circadian zeitgeber, entraining disrupted sleep–wake cycles through timed exposure to light, social interaction, and metabolic signals that synchronize the biological clock [38,39]. Exercise has been shown to phase-shift circadian rhythms in neurotypical populations, with timing-dependent effects (morning exercise advancing circadian phase; evening exercise delaying phase) that could be leveraged therapeutically in ASD populations with delayed or irregular sleep–wake patterns [40,41]. Second, physical activity may reduce anxiety-related hyperarousal through multiple pathways: acute anxiolytic effects mediated by endorphin and endocannabinoid release; chronic reductions in hypothalamic–pituitary–adrenal axis reactivity and cortisol secretion; and enhanced GABAergic neurotransmission promoting relaxation [42,43,44]. Third, physical activity may enhance homeostatic sleep drive by increasing adenosine accumulation and slow-wave sleep pressure, mechanisms that consolidate and deepen sleep architecture [7,37].

These proposed mechanisms remain speculative and require empirical testing in prospective studies. Future research should incorporate direct measurement of: (1) circadian phase via dim light melatonin onset or core body temperature rhythms; (2) hypothalamic–pituitary–adrenal axis function via serial cortisol measurements; (3) sleep architecture via polysomnography or multi-night actigraphy; (4) anxiety symptoms via validated rating scales; and (5) inflammatory markers (C-reactive protein, interleukin-6) given the bidirectional relationships between sleep, inflammation, and physical activity. Such mechanistic studies will determine which pathways mediate observed associations and may reveal patient characteristics (e.g., baseline circadian phase, anxiety severity) that predict differential treatment response, enabling more precise intervention targeting.

### 4.6. Psychiatric Comorbidity and Treatment Response

Our comorbidity-specific analyses revealed complex interactions between physical activity and concurrent psychiatric conditions. The anxiety disorder cohort demonstrated the most robust sleep improvements (−58.7% vs. −12.4%, *p* < 0.01). We propose two non-mutually exclusive explanations. First, anxiety reduction may represent a primary mechanism through which physical activity improves sleep in ASD populations. Anxiety-related hyperarousal—manifested as racing thoughts, physiological tension, and autonomic nervous system activation—directly interferes with sleep onset and maintenance [45]. By reducing anxiety through the pathways described above, physical activity may remove a primary barrier to sleep, producing particularly pronounced benefits in individuals for whom anxiety represents the dominant sleep-disrupting factor. Second, the observed pattern may reflect bidirectional causality: improved sleep may reduce daytime anxiety, which further consolidates sleep gains, creating a beneficial positive feedback loop.

However, anxiety reduction may not fully account for physical activity’s sleep benefits, as evidenced by significant improvements in non-anxious subgroups. The ADHD cohort demonstrated intermediate response patterns, while the epilepsy group showed more modest but significant benefits. These patterns suggest that physical activity operates through multiple pathways—some anxiety-dependent, others direct effects on sleep regulatory systems—with relative pathway contributions varying across comorbidity profiles. Distinguishing anxiety-mediated from sleep-direct effects will require studies incorporating validated anxiety measures, objective sleep metrics, and mediation analyses to parse direct from indirect pathways. Such mechanistic clarity has important clinical implications: if anxiety reduction proves to be a primary mechanism, combined interventions pairing physical activity with cognitive–behavioral therapy for anxiety might produce synergistic benefits exceeding either treatment alone.

### 4.7. Medication Utilization Patterns and Clinical Implications

Medication utilization patterns provided indirect but compelling evidence of sleep improvement and revealed important clinical considerations. Amphetamine use decreased more substantially in the intervention group (intervention: 19.3%→15.0%, −4.3%; control: 19.4%→16.9%, −2.5%; HR = 0.865, *p* = 0.024), suggesting that improved sleep quality may have reduced stimulant requirements in children with comorbid ASD and ADHD. This pattern has mechanistic plausibility: sleep deprivation exacerbates core ADHD symptoms including inattention, impulsivity, and hyperactivity, while sleep restoration can normalize daytime functioning sufficiently to permit stimulant dose reduction or discontinuation [46,47]. The documented improvements in insomnia (−59.81% vs. −10.71%) and sleep apnea (−62.26% vs. +9.39%) in our cohort likely normalized sleep architecture to reduce daytime symptoms that stimulants are prescribed to address. However, we note that this finding did not survive Bonferroni correction (*p* = 0.072) and should be interpreted as exploratory pending replication.

The melatonin findings require more nuanced interpretation. While both groups showed absolute decreases in melatonin use over the five-year follow-up period (intervention: 28.9%→17.1%, −11.80%; control: 27.8%→14.5%, −13.30%), the intervention group demonstrated significantly slower discontinuation when discontinuation occurred (overall HR = 1.21, 95% CI: 1.078–1.357, *p* = 0.001; anxiety subgroup HR = 1.334, *p* = 0.001). This pattern—greater melatonin persistence in the intervention group—initially appears counterintuitive if physical activity alone were sufficient to normalize sleep. We propose two competing explanations that our data cannot definitively distinguish. First, a true synergistic effect may exist whereby combining physical activity with melatonin produces superior outcomes through complementary mechanisms: physical activity providing daytime circadian signals (light exposure, metabolic activity, social zeitgebers) while melatonin delivers direct nighttime chronobiological support. Under this model, the combination therapy proves more effective than either intervention alone, leading clinicians to maintain melatonin in successfully treated patients. Second, an alternative explanation involves differential clinical monitoring intensity. Children enrolled in structured physical activity programs may receive more frequent clinical follow-up, leading to higher rates of melatonin prescription or continuation compared to control patients with less structured oversight. Under this model, melatonin persistence reflects healthcare system factors rather than therapeutic synergy. The anxiety subgroup—which demonstrated both the greatest sleep improvement (−58.7% vs. −12.4%) and highest melatonin persistence (HR = 1.334)—could exemplify either true combination therapy benefits or more intensive clinical management of high-need patients. Distinguishing these explanations requires prospective studies with standardized clinical monitoring protocols, detailed prescribing records including indication specificity and dose trajectories, and ideally a factorial design randomizing patients to physical activity alone, melatonin alone, combination therapy, or usual care. Such studies would definitively establish whether the observed pattern reflects therapeutic synergy warranting clinical implementation or an artifact of differential healthcare utilization [48].

An important methodological consideration involves potential confounding by melatonin initiation during the follow-up period. We acknowledge that our resolution rates combine effects of physical activity with potential effects of newly initiated melatonin, and we cannot isolate physical activity’s independent contribution in patients who began melatonin post-baseline. However, baseline melatonin use was balanced between groups (28.9% intervention vs. 27.8% control), and our hazard ratio analyses suggest that intervention group patients were more likely to continue melatonin rather than initiate it de novo. Future studies should stratify analyses by baseline melatonin status and use time-varying covariates or marginal structural models to appropriately handle medications initiated during follow-up, allowing clearer attribution of effects to specific interventions.

### 4.8. Implementation Challenges and Health Equity Considerations

Although our findings suggest that structured physical activity is associated with meaningful sleep improvements in autistic children and adolescents, translating these observations into equitable clinical practice presents substantial challenges that extend beyond questions of efficacy. Autistic youth face multiple interconnected barriers to physical activity participation, operating at individual, family, and structural levels. At the individual level, motor coordination impairments affect 50–90% of autistic children [27,49], creating difficulties with activities requiring precise timing, balance, or complex movement sequences. Sensory sensitivities—to noise, light, touch, or vestibular input—may render typical physical activity settings (loud gymnasiums, crowded pools, unpredictable outdoor environments) overwhelming or aversive. Social anxiety and difficulties with implicit social rules create barriers to group-based activities, while executive function challenges may impede independent exercise initiation and maintenance.

These individual-level barriers intersect with structural inequities that intensify with age. During childhood, inclusive physical activity opportunities exist through school physical education, early intervention programs, and recreational activities with lower skill requirements. However, by adolescence, organized physical activity opportunities increasingly require competitive skill levels, early specialization, and sustained participation in elite-track programs—conditions that systematically exclude many autistic youths, particularly those with motor coordination difficulties or delayed skill acquisition. Autistic adolescents report substantially lower participation in moderate-to-vigorous physical activity compared to neurotypical peers, with particular underrepresentation in team sports requiring high social demands (basketball, football, volleyball) [27,50]. Our observed age-dependent treatment response—with adolescents showing superior outcomes—may therefore reflect selection bias, as adolescents capable of accessing and sustaining physical activity interventions represent a higher-functioning subset less representative of the broader autistic population.

Access to adapted or therapeutic physical activity programs varies profoundly by geography, insurance coverage, and family socioeconomic resources. Families with higher incomes and social capital can access private adaptive sports programs, therapeutic recreation services, or individualized coaching that accommodate sensory, motor, and social needs. In contrast, families with limited resources often encounter few viable options, particularly in rural areas or communities without specialized providers. This imbalance risks exacerbating health inequities, whereby the substantial sleep benefits documented in our study accrue primarily to privileged populations while remaining inaccessible to underserved communities. The TriNetX network—comprising predominantly large healthcare organizations with integrated electronic health record systems—may further limit generalizability to community-based primary care settings serving more socioeconomically diverse populations.

Addressing these implementation gaps will require approaches extending beyond clinic-based interventions to create accessible, sustainable physical activity opportunities across the socioeconomic spectrum. We recommend that future implementation science research prioritize: (1) community-based adapted physical activity programs delivered through parks and recreation departments, YMCAs, or community centers rather than specialized medical settings; (2) school-based physical activity integration, leveraging existing infrastructure and eliminating transportation barriers; (3) family-based or caregiver-mediated interventions teaching parents to facilitate home-based physical activity; (4) low-cost, low-barrier activities (walking programs, online exercise videos, adaptive equipment lending libraries) requiring minimal specialized resources; and (5) policy interventions mandating inclusive physical education practices and requiring recreational programs receiving public funding to provide reasonable accommodations for children with disabilities. Comparative effectiveness research should examine whether community-delivered or self-directed physical activity produces sleep benefits comparable to formal clinical interventions, as broader accessibility may offset any reduction in intervention intensity or specialization. Such efforts are essential to translate efficacy findings into equitable, real-world benefits for all autistic children and adolescents, regardless of access to specialized services or family socioeconomic status.

### 4.9. Clinical Implications and Future Research Directions

Our findings support several clinical practice implications while highlighting critical knowledge gaps requiring systematic investigation. First, clinicians should consider structured physical activity as a potentially beneficial component of sleep management plans for autistic children and adolescents, particularly those with comorbid anxiety or during the adolescent period when treatment responses appear most robust. However, physical activity should complement rather than replace evidence-based sleep interventions such as behavioral sleep modification, sleep hygiene education, and judicious pharmacotherapy. Second, intervention selection should account for individual patient characteristics including motor abilities, sensory sensitivities, comorbidity profile, and family resources, with individualized rather than standardized prescriptions. Third, given the superior outcomes observed in adolescents and anxiety-comorbid patients, these populations may represent optimal targets for initial implementation efforts while mechanisms and optimal approaches for younger children are further clarified.

Future research must address several critical limitations of the current study. Prospective randomized controlled trials are essential to establish causality and should incorporate: (1) objective sleep measurement via multi-night actigraphy or polysomnography, overcoming the diagnostic code limitations that plague observational studies; (2) standardized physical activity protocols with systematic variation in dose parameters (frequency, intensity, duration, modality) to establish dose–response relationships and identify optimal prescriptions; (3) comprehensive assessment of autism severity, cognitive ability, and adaptive function to enable subgroup analyses and identify treatment moderators; (4) mechanistic assessments including circadian phase markers, anxiety ratings, inflammatory biomarkers, and anthropometric measurements to determine pathways of benefit; (5) detailed tracking of all concurrent interventions (medications, behavioral therapies, dietary modifications) to isolate physical activity effects and examine potential synergies; (6) intentional recruitment across the full autism spectrum and socioeconomic distribution, with oversampling of underserved populations to ensure findings generalize beyond well-resourced academic medical centers; and (7) extended follow-up (≥2 years) with assessment of sustained benefits and factors predicting maintenance versus relapse.

Implementation science approaches should address the translation gap between efficacy and real-world accessibility. Pragmatic trials comparing clinic-based versus community-delivered interventions, individual versus group formats, and specialized versus mainstream recreational programs will inform scalable implementation strategies. Hybrid effectiveness-implementation designs can simultaneously evaluate clinical outcomes and implementation processes, identifying barriers and facilitators to sustainable program integration. Qualitative research with autistic individuals, families, and community stakeholders can reveal preferences, priorities, and cultural considerations essential for designing acceptable and sustainable interventions.

### 4.10. Study Limitations

Several methodological considerations warrant acknowledgment. Most fundamentally, the observational nature of this retrospective cohort study precludes causal inference. Despite propensity score matching for major confounders, unmeasured factors may influence observed associations. Autism severity—encompassing symptom intensity, cognitive ability, adaptive function, and communication capacity—represents a critical unmeasured confounder, as higher-functioning individuals may demonstrate greater capacity to engage with physical activity interventions and may have fundamentally different sleep pathophysiology compared to more severely affected individuals. Objective sleep disorder severity (polysomnography findings, sleep diary data, actigraphy metrics) was unavailable, preventing us from matching on baseline sleep impairment and potentially biasing estimates if intervention and control groups differed in unmeasured severity. Socioeconomic status, though partially captured through insurance type, was incompletely measured, and substantial residual confounding by family resources, parental education, and neighborhood characteristics likely remains. Parental engagement and family functioning—powerful determinants of intervention adherence and sleep routines—were entirely unmeasured. Intervention adherence (session attendance, home practice completion, sustained participation) was not captured, creating potential for selection bias if only the most adherent patients demonstrated sleep improvements.

Substantial heterogeneity exists within our physical activity intervention cohort. Billing and procedural codes identify formal healthcare-delivered interventions but provide no information regarding modality (aerobic, resistance, aquatic, recreational), dose parameters (intensity, frequency, duration, total session number), supervision level, or intervention setting. This heterogeneity prevents establishment of dose–response relationships, identification of optimal intervention characteristics, or comparison of specific physical activity types—limitations that fundamentally constrain clinical translation. Community-based physical activities not billed through healthcare systems (recreational sports, family activities, school physical education) remain entirely uncaptured, potentially underestimating real-world physical activity exposure and misclassifying some control group participants as unexposed when they engaged in substantial physical activity outside formal healthcare contexts.

From a measurement perspective, our primary outcome—sleep disorder resolution—represents an operational definition based on absence of diagnostic codes during follow-up rather than clinically confirmed symptom resolution. This approach introduces substantial measurement error, as code absence may reflect incomplete follow-up, provider transitions, coding inconsistencies, or administrative factors rather than true clinical improvement. The propensity score matching did not account for healthcare utilization intensity or total clinical encounter frequency (excluding physical activity visits), creating potential confounding if intervention group patients received more frequent monitoring that altered sleep disorder detection, diagnosis, or coding practices independent of true clinical change. Additionally, as discussed above, polysomnography poses significant challenges for autistic individuals due to sensory sensitivities and unfamiliar environments, likely resulting in substantial underdiagnosis within our cohort and limiting outcome precision.

Statistical considerations include our application of Benjamini–Hochberg false discovery rate correction to control Type I error across multiple comparisons. Despite this adjustment, several subgroup findings (particularly in younger children) did not survive Bonferroni-adjusted significance thresholds and should be interpreted as exploratory hypotheses requiring independent replication. The large number of statistical tests (across sleep disorder subtypes, age groups, comorbidity categories, medication classes, and follow-up timepoints) increases the probability of spurious associations even after multiple testing correction. Medication utilization patterns serve as indirect proxies for sleep improvement rather than direct clinical assessments. We lack detailed prescribing information (dosages, indication specificity, temporal sequences relative to interventions, prescriber specialty) necessary to distinguish therapeutic synergy from differences in clinical monitoring intensity or prescribing practice patterns.

Generalizability requires careful consideration across multiple dimensions. The TriNetX network comprises predominantly large healthcare organizations with integrated electronic health record systems, potentially representing populations with greater healthcare access, more formalized intervention delivery, and different demographic characteristics compared to typical community-based primary care settings, safety-net systems, or rural practices. Socioeconomic and geographic diversity within the TriNetX network remains incompletely characterized, limiting our ability to assess generalizability across the full socioeconomic spectrum. Our findings reflect intervention efficacy among those who accessed and engaged with formal healthcare-delivered physical activity programs—a selected subset of the broader autistic population. Children unable to access care due to geographic, financial, or systemic barriers; those unable to tolerate or sustain interventions due to severity of symptoms; and those receiving informal physical activity support outside formal healthcare channels are systematically excluded from our analysis, potentially overestimating real-world effectiveness.

These limitations underscore the imperative for prospective randomized controlled trials incorporating objective sleep measurement (multi-night actigraphy, polysomnography), standardized physical activity protocols with documented dose parameters, comprehensive characterization of autism and sleep severity, detailed assessment of potential confounders (family functioning, socioeconomic status, concurrent interventions), and intentional recruitment across the autism spectrum and socioeconomic distribution to establish causality and inform evidence-based clinical guidelines applicable to diverse patient populations.

## 5. Conclusions

In conclusion, this study provides observational evidence suggesting that structured physical activity is associated with better sleep outcomes. The clear age-dependent patterns and comorbidity-specific responses offer valuable guidance for clinical practice while raising intriguing questions about underlying mechanisms. These findings suggest that integrating structured physical activity into treatment plans, with careful attention to individual patient characteristics and comorbidity profiles, may significantly improve sleep outcomes in this vulnerable population.

## Figures and Tables

**Table 1:** Prevalence of sleep disorders in patients with and without autism spectrum disorder by age group.

Group	Total Population	ASD	ASD with Sleep Disorders	Non-ASD	Non-ASD with Sleep Disorders
**Overall**	155,860,529	588,517	113,347	155,272,012	8,650,620
**Pediatrics**					
5–18 y	20,101,796	344,244	66,372	19,757,552	666,416
5–11 y	9,481,826	180,784	33,381	9,301,042	321,379
12–18 y	10,619,970	163,460	32,991	10,456,510	345,037

*Data is presented as number (percentage). ASD, autism spectrum disorder. Data is pooled from 132 healthcare organizations.*

**Table 2:** Baseline characteristics of children and adolescents with autism and sleep disorders before and after propensity score matching stratified by physical activity intervention status.

Characteristics	Before Matching			After Matching				
	Physical Activity	No Physical Activity	*p*-Value		Physical Activity	No Physical Activity	*p*-Value	
**Count**	3732	35,244			3709	3709		
**Demographics**								
**Age at onset**								
5–11 years	2311 (61.9%)	25,421 (72.1%)	<0.001		2306 (62.2%)	2342 (63.1%)	0.39	
12–18 years	1154 (30.9%)	7540 (21.4%)			1138 (30.7%)	1114 (30.0%)		
**Sex**								
Female	1026 (27.5%)	8551 (24.3%)	<0.001		1016 (27.4%)	988 (26.6%)	0.46	
Male	2681 (71.8%)	26,215 (74.4%)			2668 (71.9%)	2700 (72.8%)		
**Race**								
White	2372 (63.6%)	19,851 (56.3%)	<0.001		2353 (63.4%)	2426 (65.4%)	0.08	
Black or African American	589 (15.8%)	5494 (15.6%)			588 (15.9%)	559 (15.1%)		
Asian	20 (0.5%)	175 (0.5%)			20 (0.5%)	19 (0.5%)		
AIAN	88 (2.4%)	1020 (2.9%)			88 (2.4%)	93 (2.5%)		
NHPI	10 (0.3%)	60 (0.2%)			10 (0.3%)	10 (0.3%)		
Other Race	312 (8.4%)	2971 (8.4%)			310 (8.4%)	285 (7.7%)		
Unknown Race	345 (9.2%)	5673 (16.1%)			344 (9.3%)	324 (8.7%)		
**Ethnicity**								
Not Hispanic/Latino	2552 (68.4%)	22,413 (63.6%)	<0.001		2535 (68.3%)	2627 (70.8%)	0.40	
Hispanic or Latino	650 (17.4%)	6213 (17.6%)			644 (17.4%)	617 (16.6%)		
Unknown Ethnicity	530 (14.2%)	6618 (18.8%)			530 (14.3%)	465 (12.5%)		
**Comorbidities**								
Asthma	973 (26.1%)	5341 (15.2%)	<0.001		959 (25.9%)	937 (25.3%)	0.56	
Overweight	212 (5.7%)	926 (2.6%)	<0.001		205 (5.5%)	178 (4.8%)	0.16	
Obesity	697 (18.7%)	3931 (11.2%)	<0.001		682 (18.4%)	637 (17.2%)	0.17	
Mood disorders	837 (22.4%)	3511 (10%)	<0.001		820 (22.1%)	789 (21.3%)	0.38	
Anxiety disorders	1910 (51.2%)	8968 (25.4%)	<0.001		1887 (50.9%)	1896 (51.1%)	0.83	
Intellectual Disabilities	400 (10.7%)	1750 (5%)	<0.001		387 (10.4%)	400 (10.8%)	0.62	
ADHD	2129 (57%)	13,076 (37.1%)	<0.001		2107 (56.8%)	2129 (57.4%)	0.61	
Epilepsy	388 (10.4%)	2534 (7.2%)	<0.001		382 (10.3%)	390 (10.5%)	0.76	
Musculoskeletal disorders	1624 (43.5%)	6421 (18.2%)	<0.001		1602 (43.2%)	1616 (43.6%)	0.74	
**Sleep-related medications**								
Melatonin	1090 (29.2%)	4388 (12.5%)	<0.001		1072 (28.9%)	1031 (27.8%)	0.29	
Sedatives/hypnotics	1585 (42.5%)	7335 (20.8%)	<0.001		1564 (42.2%)	1571 (42.4%)	0.87	
Methylphenidate	937 (25.1%)	5504 (15.6%)	<0.001		925 (24.9%)	926 (25.0%)	0.98	
Amphetamines	727 (19.5%)	4152 (11.8%)	<0.001		716 (19.3%)	720 (19.4%)	0.91	

*Data is presented as number (percentage). Two-sided Chi-Square test was used. AIAN: American Indian or Alaska Native; NHPI: Native Hawaiian or Other Pacific Islander; ADHD: Attention-deficit hyperactivity disorders. Other medications were screened but reported in fewer than 10 patients, including zolpidem, eszopiclone, temazepam, suvorexant, modafinil, armodafinil, clonidine, and hydroxyzine.*

**Table 3:** Resolution rates of sleep disorder subtypes by physical activity intervention status before and after matching.

Type	Follow-Up Time	Before Matching			After Matching		
		Physical Activity	No Physical Activity	*p*-Value	Physical Activity	No Physical Activity	*p*-Value
Insomnia	After 1 y	−60.06	16.37	**0.001**	−59.81	−10.71	**0.042**
	After 5 y	−43.54	57.59	**<0.001**	−43.34	18.21	0.23
Hypersomnia	After 1 y	−21.71	29.04	0.06	−60.16	−18.81	0.22
	After 5 y	−30.23	86.14	**<0.001**	−32.03	−5.94	0.82
Circadian rhythm sleep disorders	After 1 y	−72.09	−20.82	0.30	−71.65	−19.12	**0.035**
	After 5 y	−48.06	25.10	0.91	−47.24	11.76	0.45
Sleep apnea	After 1 y	−62.30	−8.90	**0.006**	−62.26	9.39	0.004 *
	After 5 y	−47.79	12.61	0.88	−47.69	8.82	**0.027**
Parasomnia	After 1 y	−83.56	−21.94	**0.040**	−83.33	−1.79	**0.016**
	After 5 y	−64.38	2.16	0.48	−63.89	−14.29	**0.048**

Data is presented as number (percentage). Two-sided Chi-Square test was used. Resolution rate was estimated using the following formula: [(Cases at follow-up − Initial cases)/Initial cases] × 100%. Negative values indicate improvement from baseline; Positive values indicate worsening from baseline. Bold values indicate statistically significant. Benjamini–Hochberg false discovery rate (FDR) correction was employed to control for Type I errors. We examined five specific sleep disorder types at two follow-up timepoints (1-year and 5-year), comprising 10 statistical tests. * Significant values after multiple comparison test.

**Table 4:** Resolution rates of sleep disorder after matching stratified by age group.

Subgroup	Type of Sleep Disorders	1-Year Follow-Up			5-Year Follow-Up		
		Physical Activity	No Physical Activity	*p*-Value	Physical Activity	No Physical Activity	*p*-Value
5–11 years	Sleep disorders	−56.30	−7.05	**0.036**	−60.61	−49.92	0.49
	Insomnia	−52.00	−27.43	**0.025**	−41.65	−6.64	0.06
	Hypersomnia	−28.57	19.47	0.79	−9.52	2.63	0.96
	Circadian rhythm sleep disorders	−50.00	−13.64	**0.041**	−59.62	−16.36	**0.007**
	Sleep apnea	−64.22	−10.00	0.78	−63.20	−52.67	0.45
	Parasomnia	−73.33	−16.15	**0.036**	−73.33	−12.31	0.56
12–18 years	Sleep disorders	−82.91	−21.00	0.003 *	−80.06	−7.14	<0.001 *
	Insomnia	−68.77	−22.43	**0.032**	−60.89	−36.45	**0.006**
	Hypersomnia	−10.00	18.00	0.13	−14.62	24.00	0.31
	Circadian rhythm sleep disorders	−44.44	−37.84	0.64	−22.22	−24.32	1.00
	Sleep apnea	−80.42	−14.46	**0.036**	−81.47	20.46	<0.001 *
	Parasomnia	−50.00	−53.33	1.00	−53.85	−56.67	1.00

Data is presented as number (percentage). Two-sided Chi-Square test was used. Resolution rate was estimated using the following formula: [(Cases at follow-up − Initial cases)/Initial cases] × 100%. Negative values indicate improvement from baseline; Positive values indicate worsening from baseline. Bold values indicate statistically significant. Benjamini–Hochberg false discovery rate (FDR) correction was employed to control for Type I errors. Age-stratified analyses controlled for 12 statistical tests per age group (6 sleep disorders × 2 timepoints), with Bonferroni-corrected α = 0.00417 per age stratum. * Significant values after multiple comparison test.

**Table 5:** Resolution rates of sleep disorders by comorbidity after matching.

Type	ADHD (n = 4896)	Anxiety (n = 4010)	Epilepsy (n = 880)
**Sleep disorders**			
1 y	−54.2 vs. −8.3 **	−58.7 vs. −12.4 **	−45.6 vs. −2.8 *
5 y	−46.8 vs. +5.4 **	−51.2 vs. +3.9 **	−38.9 vs. +8.7 *
**Insomnia**			
1 y	−55.4 vs. −15.2 *	−61.8 vs. −18.6 **	−48.2 vs. −8.9 *
5 y	−41.2 vs. +12.4 *	−45.6 vs. +15.8 **	−35.4 vs. +20.3
**Hypersomnia**			
1 y	−58.4 vs. −20.3	−62.5 vs. −22.4	−45.2 vs. −15.6
5 y	−30.6 vs. −8.2	−35.8 vs. −10.4	−28.4 vs. −4.8
**Circadian rhythm sleep disorders**			
1 y	−68.4 vs. −22.3 *	−73.8 vs. −25.6 **	−55.4 vs. −12.8 *
5 y	−45.6 vs. +8.9	−50.2 vs. +10.4 *	−40.2 vs. +15.6
**Sleep apnea**			
1 y	−60.4 vs. +5.8 **	−65.8 vs. +8.9 **	−52.4 vs. +12.6 *
5 y	−45.8 vs. +6.4 *	−50.2 vs. +10.2 **	−42.8 vs. +15.4 *
**Parasomnia**			
1 y	−80.4 vs. −5.2 **	−85.6 vs. −8.4 **	−75.4 vs. +2.8 **
5 y	−60.2 vs. −18.4 *	−65.8 vs. −20.6 *	−55.4 vs. −10.6 *

Values represent resolution rates (%) in physical activity versus no physical activity groups. Resolution rate was estimated using the following formula: [(Cases at follow-up − Initial cases)/Initial cases] × 100%. Negative values indicate improvement from baseline; Positive values indicate worsening from baseline. * p < 0.05, ** p < 0.01.

**Table 6:** Overall cohort medication utilization changes (N = 7418).

Medication/Group	Baseline	Follow-Up	Absolute Change	Change vs. Control	*p*-Value	Hazard Ratio	95% CI
**Melatonin**							
Physical Activity	28.90%	17.10%	−11.80%	2.60%	0.001 *	1.21	(1.078, 1.357)
No Physical Activity	27.80%	14.50%	−13.30%	Reference			
**Methylphenidate**							
Physical Activity	24.90%	19.20%	−5.70%	−1.30%	0.17	0.928	(0.838, 1.028)
No Physical Activity	25.00%	20.50%	−4.50%	Reference			
**Amphetamines**							
Physical Activity	19.30%	15.00%	−4.30%	−1.90%	**0.024**	0.865	(0.771, 0.969)
No Physical Activity	19.40%	16.90%	−2.50%	Reference			

Values represent medication utilization prevalence (%) at baseline and follow-up in physical activity versus no physical activity groups. Absolute Change = (Follow-up% − Baseline%); negative values indicate decreased medication use; positive values indicate increased use. Hazard Ratio (HR) represents the relative rate of medication discontinuation in the physical activity group compared to controls. HR > 1 indicates higher discontinuation rate (faster decline) in the physical activity group. HR < 1 indicates lower discontinuation rate (slower decline) in the physical activity group. CI: confidence interval. Bold values indicate statistically significant. Benjamini–Hochberg false discovery rate (FDR) correction was employed to control for Type I errors. * Significant values after multiple comparison test. Melatonin meeting Bonferroni-corrected significance (Bonf p = 0.003).

**Table 7:** Comorbidity-specific analysis.

Medication/Group	Baseline	Follow-Up	Absolute Change	Change vs. Control	*p*-Value	Hazard Ratio	95% CI
**ADHD (N = 4896)**							
**Melatonin**							
Physical Activity	33.00%	19.40%	−13.60%	3.70%	0.001 *	1.303	(1.139, 1.491)
No Physical Activity	32.60%	15.70%	−16.90%	Reference			
**Methylphenidate**							
Physical Activity	37.30%	27.40%	−9.90%	−0.10%	0.85	1.003	(0.902, 1.116)
No Physical Activity	37.90%	27.60%	−10.30%	Reference			
**Amphetamines**							
Physical Activity	28.90%	21.70%	−7.20%	−1.30%	0.21	0.937	(0.832, 1.054)
No Physical Activity	29.20%	23.20%	−6.00%	Reference			
**Anxiety (N = 4010)**							
**Melatonin**							
Physical Activity	32.90%	19.20%	−13.70%	4.00%	0.001 *	1.334	(1.147, 1.550)
No Physical Activity	31.00%	15.20%	−15.80%	Reference			
**Methylphenidate**							
Physical Activity	32.60%	23.00%	−9.60%	0.10%	0.94	1.032	(0.907, 1.174)
No Physical Activity	34.00%	22.90%	−11.10%	Reference			
**Amphetamines**							
Physical Activity	25.60%	18.30%	−7.30%	−0.90%	0.44	0.955	(0.827, 1.102)
No Physical Activity	26.00%	19.20%	−6.80%	Reference			
**Epilepsy (N = 880)**							
**Melatonin**							
Physical Activity	33.60%	23.60%	−10.00%	3.40%	0.22	1.135	(0.855, 1.507)
No Physical Activity	34.50%	20.20%	−14.30%	Reference			
**Methylphenidate**							
Physical Activity	22.00%	17.30%	−4.70%	2.10%	0.41	1.118	(0.805, 1.553)
No Physical Activity	21.10%	15.20%	−5.90%	Reference			
**Amphetamines**							
Physical Activity	13.40%	9.10%	−4.30%	−4.10%	0.054	0.633	(0.423, 0.948)
No Physical Activity	13.00%	13.20%	0.20%	Reference			

Values represent medication utilization prevalence (%) at baseline and follow-up in comorbidity-specific subgroups with physical activity versus no physical activity groups. Absolute Change = (Follow-up% − Baseline%); negative values indicate decreased medication use; positive values indicate increased medication use. Hazard Ratio (HR) represents the relative rate of medication discontinuation in the physical activity group compared to controls within each comorbidity subgroup. HR > 1 indicates higher discontinuation rate (faster decline) in the physical activity group; HR < 1 indicates lower discontinuation rate (slower decline). 95%CI: confidence interval. Bold values indicate statistically significant. Benjamini–Hochberg false discovery rate (FDR) correction was employed to control for Type I errors. * Significant values after multiple comparison test. Melatonin persistent in both ADHD & Anxiety patients were significant (Bonferroni-adjusted p-values = 0.009).

## Data Availability

Data used in this study were obtained from the TriNetX research network and are not publicly available. Access requires institutional affiliation and a data use agreement with TriNetX (https://www.trinetx.com/, accessed on 29 November 2024). Our data were obtained from the TriNetX database and are available at (https://trinetx.com/, accessed on 29 November 2024) with the permission of the TriNetX database authority. Researchers interested in accessing TriNetX data can contact the TriNetX research team directly.

## Supplementary Materials

The following supporting information can be downloaded at: https://www.mdpi.com/article/10.3390/children13010048/s1, Table S1: Codes used for inclusion criteria; Table S2: Codes used for exclusion criteria; Table S3: Codes for outcomes (new-onset sleep disorders); Table S4: Time-to-event analysis of sleep disorders by subtype after matching; Table S5: Baseline characteristics of cohorts aged 5 to 11 years; Table S6: Baseline characteristics of cohorts aged 12 to 18 years; Table S7: Baseline characteristics of cohorts with attention-deficit hyperactivity disorders; Table S8: Baseline characteristics of cohorts with anxiety; Table S9: Baseline characteristics of cohorts with epilepsy.
