# Supplementary material for: Breaking the Cycle: Impact of Physical Activity on Sleep Disorders in Autism—A Five-Year Longitudinal Analysis
Source: Children (Basel). 2025 Dec 30;13(1):48. doi: 10.3390/children13010048 (PMC12840406; doi:10.3390/children13010048)
Supplement: Supplementary file 1 [file children-13-00048-s001.zip › children-4030064-supplementary.pdf]

Breaking the Cycle: Impact of Physical Activity on Sleep Dis-orders in Autism - A Five-Year Longitudinal Analysis

Supplementary Table S1. Codes used for inclusion criteria.

Criteria	Category	Code	Description
Age	Demographics	Age	Age (at most 18 years (most recent occurrence))
Autism	Diagnosis	UMLS:ICD10CM:F84.0	Autistic disorder (between 5 and 18 years old at event)
Sleep disorders	Diagnosis	UMLS:ICD10CM:G47	Sleep disorders
Physical activity	Procedure	UMLS:SNOMED:819961005	Physical activity guidance
	Procedure	UMLS:CPT:97530	Therapeutic activities, direct (one-on-one) patient contact (use of dynamic activities to improve functional performance), each 15 minutes
	Procedure	UMLS:CPT:97112	Therapeutic procedure, 1 or more areas, each 15 minutes; neuromuscular reeducation of movement, balance, coordination, kinesthetic sense, posture, and/or proprioception for sitting and/or standing activities
	Procedure	UMLS:CPT:97150	Therapeutic procedure(s), group (2 or more individuals)
	Procedure	UMLS:SNOMED:91251008	Physical therapy procedure
	Procedure	UMLS:CPT:1013483	Physical Medicine and Rehabilitation Evaluations
	Procedure	UMLS:CPT:1013510	Physical Medicine and Rehabilitation Therapeutic Procedures
	Procedure	UMLS:SNOMED:229065009	Exercise therapy
	Procedure	UMLS:ICD10PCS:F	Physical Rehabilitation and Diagnostic Audiology
	Procedure	UMLS:CPT:97116	Therapeutic procedure, 1 or more areas, each 15 minutes; gait training (includes stair climbing)
	Procedure	UMLS:CPT:1013490	Physical Medicine and Rehabilitation Modalities
	Procedure	UMLS:SNOMED:108305003	Physical rehabilitation therapy procedure
	Procedure	UMLS:CPT:1013538	Physical Medicine and Rehabilitation Tests and Measurements
	Diagnosis	UMLS:ICD10CM:Y93.A3	Activity, aerobic and step exercise
	Procedure	UMLS:SNOMED:66500008	Physical medicine procedure
	Procedure	UMLS:HCPCS:S9451	Exercise classes, non-physician provider, per session
	Procedure	UMLS:ICD10PCS:F07M6FZ	Therapeutic Exercise Treatment of Musculoskeletal System - Whole Body using Assistive, Adaptive, Supportive or Protective Equipment
	Procedure	UMLS:ICD10PCS:F07M6YZ	Therapeutic Exercise Treatment of Musculoskeletal System - Whole Body using Other Equipment
	Procedure	UMLS:ICD10PCS:F07M6ZZ	Therapeutic Exercise Treatment of Musculoskeletal System - Whole Body
	Procedure	UMLS:CPT:97110	Therapeutic procedure, 1 or more areas, each 15 minutes; therapeutic exercises to develop

			strength and endurance, range of motion and flexibility
	Procedure	UMLS:ICD10PCS:F07L6YZ	Therapeutic Exercise Treatment of Musculoskeletal System - Lower Back / Lower Extremity using Other Equipment
	Procedure	UMLS:ICD10PCS:F07K6ZZ	Therapeutic Exercise Treatment of Musculoskeletal System - Upper Back / Upper Extremity
	Procedure	UMLS:ICD10PCS:F07J6YZ	Therapeutic Exercise Treatment of Musculoskeletal System - Head and Neck using Other Equipment
	Procedure	UMLS:ICD10PCS:F07L6ZZ	Therapeutic Exercise Treatment of Musculoskeletal System - Lower Back / Lower Extremity
	Procedure	UMLS:ICD10PCS:F07K6YZ	Therapeutic Exercise Treatment of Musculoskeletal System - Upper Back / Upper Extremity using Other Equipment
	Procedure	UMLS:ICD10PCS:F07L6FZ	Therapeutic Exercise Treatment of Musculoskeletal System - Lower Back / Lower Extremity using Assistive, Adaptive, Supportive or Protective Equipment

Supplementary Table S2. Codes used for exclusion criteria.

Criteria	Category	Code	Description
Chromosomal abnormalities	Diagnosis	UMLS:ICD10CM:Q90-Q99	Chromosomal abnormalities, not elsewhere classified
Paralysis	Diagnosis	UMLS:ICD10CM:G80-G83	Cerebral palsy and other paralytic syndromes
Congenital abnormalities	Diagnosis	UMLS:ICD10CM:Q24	Other congenital malformations of heart
	Diagnosis	UMLS:ICD10CM:Q20	Congenital malformations of cardiac chambers and connections
Neoplasm	Diagnosis	UMLS:ICD10CM:C00-D49	Neoplasms

Supplementary Table S3. Codes for outcomes (new-onset sleep disorders).

Criteria	Category	Code	Description
Sleep disorders	Diagnosis	UMLS:ICD10CM:G47	Sleep disorders
Insomnia	Diagnosis	UMLS:ICD10CM:G47.0	Insomnia
	Diagnosis	UMLS:ICD10CM:F51.05	Insomnia due to other mental disorder
Sleep apnea	Diagnosis	UMLS:ICD10CM:G47.3	Sleep apnea
Hypersomnia	Diagnosis	UMLS:ICD10CM:G47.1	Hypersomnia
Circadian rhythm sleep disorders	Diagnosis	UMLS:ICD10CM:G47.2	Circadian rhythm sleep disorders
Parasomnia	Diagnosis	UMLS:ICD10CM:G47.5	Parasomnia
Narcolepsy and cataplexy	Diagnosis	UMLS:ICD10CM:G47.4	Narcolepsy and cataplexy
Prescription of sleep medications	Medication	NLM:RXNORM:6711	Melatonin
	Medication	NLM:RXNORM:6901	methylphenidate
	Medication	NLM:VA:CN801	Amphetamines

Supplementary Table S4. Time-to-event analysis of sleep disorders by subtype after matching.

Sleep disorder type	1 year		5 years	
	HR	95%CI	HR	95%CI
Insomnia	**0.84**	**(0.77, 0.92)**	0.92	(0.85, 1.00)
Hypersomnia	0.87	(0.73, 1.04)	0.94	(0.80, 1.11)
Circadian rhythm sleep disorders	**0.78**	**(0.65, 0.94)**	0.89	(0.75, 1.06)
Sleep apnea	**0.81**	**(0.73, 0.90)**	**0.87**	**(0.79, 0.96)**
Parasomnia	**0.76**	**(0.63, 0.92)**	**0.85**	**(0.72, 0.99)**

Cox regression test was employed. HR: hazards ratio; CI: confidence interval. Bold values indicate statistically significant.

Supplementary Table S5. Baseline characteristics of cohorts aged 5 to 11 years.

Characteristics	Before matching			After matching		
	**Physical activity**	**No physical activity**	**p-value**	**Physical activity**	**No physical activity**	**p-value**
Count	1,355	14,743		1,341	1,341	
**Demographics**						
Sex						
Female	321 (23.7%)	3587 (24.3%)	0.59	317 (23.6%)	305 (22.7%)	0.58
Male	1028 (75.9%)	10982 (74.5%)		1018 (75.9%)	1031 (76.9%)	
Race						
White	778 (57.4%)	7690 (52.2%)	<0.001	768 (57.3%)	804 (60%)	0.16
Black or African American	265 (19.6%)	2651 (18%)		263 (19.6%)	237 (17.7%)	
Asian	10 (0.7%)	78 (0.5%)		10 (0.7%)	10 (0.7%)	
AIAN	34 (2.5%)	455 (3.1%)		34 (2.5%)	44 (3.3%)	
NHPI	10 (0.7%)	29 (0.2%)		10 (0.7%)	10 (0.7%)	
Other Race	130 (9.6%)	1315 (8.9%)		129 (9.6%)	128 (9.5%)	
Unknown Race	139 (10.3%)	2525 (17.1%)		138 (10.3%)	121 (9%)	
Ethnicity						
Not Hispanic or Latino	870 (64.2%)	9007 (61.1%)	0.024	860 (64.1%)	910 (67.9%)	0.91
Hispanic or Latino	284 (21%)	2932 (19.9%)		281 (21%)	229 (17.1%)	
Unknown Ethnicity	201 (14.8%)	2804 (19%)		200 (14.9%)	202 (15.1%)	
**Comorbidities**						
Asthma	328 (24.2%)	2344 (15.9%)	<0.001	321 (23.9%)	326 (24.3%)	0.82
Overweight	72 (5.3%)	340 (2.3%)	<0.001	68 (5.1%)	60 (4.5%)	0.47
Obesity	201 (14.8%)	1414 (9.6%)	<0.001	192 (14.3%)	169 (12.6%)	0.19
Mood disorders	107 (7.9%)	492 (3.3%)	<0.001	104 (7.8%)	85 (6.3%)	0.15
Anxiety disorders	538 (39.7%)	2611 (17.7%)	<0.001	524 (39.1%)	532 (39.7%)	0.75
Intellectual Disabilities	95 (7%)	497 (3.4%)	<0.001	91 (6.8%)	91 (6.8%)	1.00
ADHD	698 (51.5%)	4624 (31.4%)	<0.001	685 (51.1%)	719 (53.6%)	0.19
Epilepsy	121 (8.9%)	932 (6.3%)	<0.001	120 (8.9%)	114 (8.5%)	0.68
Musculoskeletal disorders	506 (37.3%)	2683 (18.2%)	<0.001	493 (36.8%)	485 (36.2%)	0.75
**Sleep-related medications**						
Melatonin	366 (27%)	1840 (12.5%)	<0.001	357 (26.6%)	350 (26.1%)	0.76
Sedatives/hypnotics	629 (46.4%)	3445 (23.4%)	<0.001	616 (45.9%)	616 (45.9%)	1.00
Methylphenidate	324 (23.9%)	1848 (12.5%)	<0.001	314 (23.4%)	334 (24.9%)	0.37
Amphetamines	217 (16%)	1213 (8.2%)	<0.001	210 (15.7%)	210 (15.7%)	1.00

Data is presented as number (percentage). Two-sided Chi-Square test was used. AIAN: American Indian or Alaska Native; NHPI: Native Hawaiian or Other Pacific Islander; ADHD: Attention-deficit hyperactivity disorders.

Supplementary Table S6. Baseline characteristics of cohorts aged 12 to 18 years.

Characteristics	Before matching			After matching		
	Physical activity	No physical activity	p-value	Physical activity	No physical activity	p-value
Count	830	9,341		817	817	
**Demographics**						
Sex						
Female	323 (38.9%)	2522 (27%)	<0.001	313 (38.3%)	313 (38.3%)	1.0
Male	495 (59.6%)	6658 (71.3%)		492 (60.2%)	493 (60.3%)	
Race						
White	562 (67.7%)	5518 (59.1%)	<0.001	552 (67.6%)	571 (69.9%)	0.31
Black or African American	118 (14.2%)	1274 (13.6%)		116 (14.2%)	96 (11.8%)	
Asian	10 (1.2%)	48 (0.5%)		10 (1.2%)	10 (1.2%)	
AIAN	14 (1.7%)	297 (3.2%)		14 (1.7%)	14 (1.7%)	
NHPI	10 (1.2%)	14 (0.1%)		10 (1.2%)	10 (1.2%)	
Other Race	73 (8.8%)	810 (8.7%)		72 (8.8%)	72 (8.8%)	
Unknown Race	53 (6.4%)	1380 (14.8%)		53 (6.5%)	53 (6.5%)	
Ethnicity						
Not Hispanic or Latino	578 (69.6%)	6175 (66.1%)	0.039	573 (70.1%)	606 (74.2%)	0.07
Hispanic or Latino	112 (13.5%)	1361 (14.6%)		109 (13.3%)	99 (12.1%)	
Unknown Ethnicity	140 (16.9%)	1805 (19.3%)		135 (16.5%)	112 (13.7%)	
**Comorbidities**						
Asthma	238 (28.7%)	1520 (16.3%)	<0.001	232 (28.4%)	219 (26.8%)	0.47
Overweight	58 (7%)	438 (4.7%)	0.003	55 (6.7%)	46 (5.6%)	0.36
Obesity	235 (28.3%)	1896 (20.3%)	<0.001	228 (27.9%)	205 (25.1%)	0.20
Mood disorders	408 (49.2%)	2422 (25.9%)	<0.001	397 (48.6%)	404 (49.4%)	0.73
Anxiety disorders	601 (72.4%)	4425 (47.4%)	<0.001	588 (72%)	604 (73.9%)	0.37
Intellectual Disabilities	132 (15.9%)	1004 (10.7%)	<0.001	127 (15.5%)	143 (17.5%)	0.29
ADHD	513 (61.8%)	4717 (50.5%)	<0.001	504 (61.7%)	496 (60.7%)	0.69
Epilepsy	116 (14%)	894 (9.6%)	<0.001	111 (13.6%)	107 (13.1%)	0.77
Musculoskeletal disorders	466 (56.1%)	2390 (25.6%)	<0.001	453 (55.4%)	464 (56.8%)	0.58
**Sleep-related medications**						
Melatonin	313 (37.7%)	1650 (17.7%)	<0.001	304 (37.2%)	283 (34.6%)	0.28
Sedatives/hypnotics	369 (44.5%)	2396 (25.7%)	<0.001	359 (43.9%)	348 (42.6%)	0.58
Methylphenidate	196 (23.6%)	2081 (22.3%)	0.38	194 (23.7%)	192 (23.5%)	0.91
Amphetamines	167 (20.1%)	1762 (18.9%)	0.38	166 (20.3%)	148 (18.1%)	0.26

Data is presented as number (percentage). Two-sided Chi-Square test was used. AIAN: American Indian or Alaska Native; NHPI: Native Hawaiian or Other Pacific Islander; ADHD: Attention-deficit hyperactivity disorders

Supplementary Table S7. Baseline characteristics of cohorts with attention-deficit hyperactivity disorders.

Characteristics	Before matching			After matching		
	**Physical activity**	**No physical activity**	**p-value**	**Physical activity**	**No physical activity**	**p-value**
Count	2,468	19,840		2,448	2,448	
**Demographics**						
Age at onset						
5 - 11 years	1438 (58.3%)	13393 (67.5%)	<0.001	1436 (58.7%)	1474 (60.2%)	0.27
12 - 18 years	836 (33.9%)	4900 (24.7%)		818 (33.4%)	791 (32.3%)	
Sex						
Female	625 (25.3%)	4402 (22.2%)	<0.001	614 (25.1%)	608 (24.8%)	0.84
Male	1825 (73.9%)	15166 (76.4%)		1816 (74.2%)	1824 (74.5%)	
Race						
White	1643 (66.6%)	12038 (60.7%)	<0.001	1629 (66.5%)	1677 (68.5%)	0.14
Black or African American	373 (15.1%)	2981 (15%)		371 (15.2%)	372 (15.2%)	
Asian	13 (0.5%)	98 (0.5%)		13 (0.5%)	16 (0.7%)	
AIAN	40 (1.6%)	419 (2.1%)		39 (1.6%)	36 (1.5%)	
NHPI	10 (0.4%)	22 (0.1%)		10 (0.4%)	10 (0.4%)	
Other Race	182 (7.4%)	1468 (7.4%)		180 (7.4%)	148 (6%)	
Unknown Race	214 (8.7%)	2814 (14.2%)		213 (8.7%)	196 (8%)	
Ethnicity						
Not Hispanic or Latino	1741 (70.5%)	13383 (67.5%)	0.002	1726 (70.5%)	1776 (72.5%)	0.11
Hispanic or Latino	370 (15%)	2825 (14.2%)		367 (15%)	346 (14.1%)	
Unknown Ethnicity	357 (14.5%)	3632 (18.3%)		355 (14.5%)	326 (13.3%)	
**Comorbidities**						
Asthma	677 (27.4%)	3249 (16.4%)	<0.001	666 (27.2%)	650 (26.6%)	0.61
Overweight	146 (5.9%)	596 (3%)	<0.001	142 (5.8%)	135 (5.5%)	0.67
Obesity	466 (18.9%)	2322 (11.7%)	<0.001	456 (18.6%)	441 (18%)	0.58
Mood disorders	702 (28.4%)	2983 (15%)	<0.001	685 (28%)	631 (25.8%)	0.08
Anxiety disorders	1461 (59.2%)	7205 (36.3%)	<0.001	1441 (58.9%)	1446 (59.1%)	0.88
Intellectual Disabilities	280 (11.3%)	1204 (6.1%)		271 (11.1%)	265 (10.8%)	
Epilepsy	241 (9.8%)	1449 (7.3%)	<0.001	238 (9.7%)	224 (9.2%)	0.49
Musculoskeletal disorders	1081 (43.8%)	3917 (19.7%)	<0.001	1063 (43.4%)	1086 (44.4%)	0.51
**Sleep-related medications**						
Melatonin	826 (33.5%)	3197 (16.1%)	<0.001	809 (33%)	797 (32.6%)	0.72
Sedatives/hypnotics	1016 (41.2%)	4381 (22.1%)	<0.001	999 (40.8%)	963 (39.3%)	0.29
Methylphenidate	921 (37.3%)	5444 (27.4%)	<0.001	912 (37.3%)	929 (37.9%)	0.62
Amphetamines	717 (29.1%)	4071 (20.5%)	<0.001	708 (28.9%)	715 (29.2%)	0.83

Data is presented as number (percentage). Two-sided Chi-Square test was used. AIAN: American Indian or Alaska Native; NHPI: Native Hawaiian or Other Pacific Islander.

Supplementary Table S8. Baseline characteristics of cohorts with anxiety.

Characteristics	Before matching			After matching		
	**Physical activity**	**No physical activity**	**p-value**	**Physical activity**	**No physical activity**	**p-value**
Count	2,035	12,967		2,005	2,005	
**Demographics**						
Age at onset						
5 - 11 years	962 (47.3%)	6925 (53.4%)	<0.001	954 (47.6%)	1000 (49.9%)	0.15
12 - 18 years	887 (43.6%)	4822 (37.2%)		866 (43.2%)	829 (41.3%)	
Sex						
Female	671 (33%)	3724 (28.7%)	<0.001	657 (32.8%)	648 (32.3%)	0.76
Male	1346 (66.1%)	9001 (69.4%)		1330 (66.3%)	1339 (66.8%)	
Race						
White	1460 (71.7%)	8737 (67.4%)	<0.001	1437 (71.7%)	1471 (73.4%)	0.23
Black or African American	233 (11.4%)	1321 (10.2%)		230 (11.5%)	225 (11.2%)	
Asian	10 (0.5%)	63 (0.5%)		10 (0.5%)	11 (0.5%)	
AIAN	26 (1.3%)	290 (2.2%)		25 (1.2%)	27 (1.3%)	
NHPI	10 (0.5%)	18 (0.1%)		10 (0.5%)	10 (0.5%)	
Other Race	151 (7.4%)	964 (7.4%)		148 (7.4%)	136 (6.8%)	
Unknown Race	150 (7.4%)	1574 (12.1%)		150 (7.5%)	131 (6.5%)	
Ethnicity						
Not Hispanic or Latino	1465 (72%)	9065 (69.9%)	0.06	1441 (71.9%)	1480 (73.8%)	0.17
Hispanic or Latino	292 (14.3%)	1741 (13.4%)		288 (14.4%)	264 (13.2%)	
Unknown Ethnicity	278 (13.7%)	2161 (16.7%)		276 (13.8%)	261 (13%)	
**Comorbidities**						
Asthma	544 (26.7%)	2135 (16.5%)	<0.001	524 (26.1%)	537 (26.8%)	0.64
Overweight	138 (6.8%)	441 (3.4%)	<0.001	131 (6.5%)	111 (5.5%)	0.19
Obesity	418 (20.5%)	1742 (13.4%)	<0.001	403 (20.1%)	352 (17.6%)	0.04
Mood disorders	724 (35.6%)	2930 (22.6%)	<0.001	704 (35.1%)	664 (33.1%)	0.18
Intellectual Disabilities	219 (10.8%)	854 (6.6%)	<0.001	209 (10.4%)	197 (9.8%)	0.53
ADHD	1399 (68.7%)	7565 (58.3%)	<0.001	1374 (68.5%)	1378 (68.7%)	0.89
Epilepsy	183 (9%)	857 (6.6%)	<0.001	178 (8.9%)	146 (7.3%)	0.06
Musculoskeletal disorders	999 (49.1%)	3050 (23.5%)	<0.001	972 (48.5%)	997 (49.7%)	0.43
**Sleep-related medications**						
Melatonin	682 (33.5%)	2241 (17.3%)	<0.001	659 (32.9%)	622 (31%)	0.21
Sedatives/hypnotics	897 (44.1%)	3204 (24.7%)	<0.001	870 (43.4%)	838 (41.8%)	0.31
Methylphenidate	664 (32.6%)	3487 (26.9%)	<0.001	654 (32.6%)	682 (34%)	0.35
Amphetamines	524 (25.7%)	2635 (20.3%)	<0.001	514 (25.6%)	522 (26%)	0.77

Data is presented as number (percentage). Two-sided Chi-Square test was used. AIAN: American Indian or Alaska Native; NHPI: Native Hawaiian or Other Pacific Islander; ADHD: Attention-deficit hyperactivity disorders.

Supplementary Table S9. Baseline characteristics of cohorts with epilepsy.

Characteristics	Before matching			After matching		
	Physical activity	No physical activity	p-value	Physical activity	No physical activity	p-value
Count	461	3,471		440	440	
**Demographics**						
Age at onset						
5 - 11 years	252 (54.7%)	2181 (62.8%)	0.001	247 (56.1%)	255 (58%)	0.59
12 - 18 years	175 (38%)	998 (28.8%)		161 (36.6%)	157 (35.7%)	
Sex						
Female	129 (28%)	950 (27.4%)	0.78	124 (28.2%)	127 (28.9%)	0.82
Male	331 (71.8%)	2488 (71.7%)		315 (71.6%)	312 (70.9%)	
Race						
White	284 (61.6%)	2031 (58.5%)	0.21	270 (61.4%)	249 (56.6%)	0.15
Black or African American	82 (17.8%)	545 (15.7%)		80 (18.2%)	86 (19.5%)	
Asian	10 (2.2%)	21 (0.6%)		10 (2.3%)	10 (2.3%)	
AIAN	10 (2.2%)	119 (3.4%)		10 (2.3%)	16 (3.6%)	
NHPI	10 (2.2%)	10 (0.3%)		10 (2.3%)	10 (2.3%)	
Other Race	37 (8%)	251 (7.2%)		35 (8%)	38 (8.6%)	
Unknown Race	41 (8.9%)	499 (14.4%)		40 (9.1%)	44 (10%)	
Ethnicity						
Not Hispanic or Latino	325 (70.5%)	2243 (64.6%)	0.013	309 (70.2%)	309 (70.2%)	1.00
Hispanic or Latino	92 (20%)	648 (18.7%)		87 (19.8%)	84 (19.1%)	
Unknown Ethnicity	44 (9.5%)	580 (16.7%)		44 (10%)	47 (10.7%)	
**Comorbidities**						
Asthma	146 (31.7%)	615 (17.7%)	<0.001	131 (29.8%)	138 (31.4%)	0.61
Overweight	35 (7.6%)	109 (3.1%)	<0.001	31 (7%)	30 (6.8%)	0.89
Obesity	99 (21.5%)	440 (12.7%)	<0.001	89 (20.2%)	91 (20.7%)	0.87
Mood disorders	97 (21%)	333 (9.6%)	<0.001	85 (19.3%)	84 (19.1%)	0.93
Anxiety disorders	224 (48.6%)	898 (25.9%)	<0.001	205 (46.6%)	205 (46.6%)	1.00
Intellectual Disabilities	126 (27.3%)	467 (13.5%)	<0.001	110 (25%)	111 (25.2%)	0.94
ADHD	248 (53.8%)	1383 (39.8%)	<0.001	233 (53%)	243 (55.2%)	0.50
Musculoskeletal disorders	224 (48.6%)	810 (23.3%)	<0.001	207 (47%)	204 (46.4%)	0.84
**Sleep-related medications**						
Melatonin	165 (35.8%)	659 (19%)	<0.001	148 (33.6%)	152 (34.5%)	0.78
Sedatives/hypnotics	378 (82%)	2023 (58.3%)	<0.001	358 (81.4%)	352 (80%)	0.61
Methylphenidate	104 (22.6%)	545 (15.7%)	<0.001	97 (22%)	93 (21.1%)	0.74
Amphetamines	61 (13.2%)	402 (11.6%)	0.302	59 (13.4%)	57 (13%)	0.84

Data is presented as number (percentage). Two-sided Chi-Square test was used. AIAN: American Indian or Alaska Native; NHPI: Native Hawaiian or Other Pacific Islander; ADHD: Attention-deficit hyperactivity disorders.
